# Urban and Rural Differences in the Efficacy of a Mobile Health Depression Treatment for Young Adults

**DOI:** 10.3390/ijerph21121572

**Published:** 2024-11-26

**Authors:** Jeremy Mennis, J. Douglas Coatsworth, Michael Russell, Nikola Zaharakis, Aaron R. Brown, Michael J. Mason

**Affiliations:** 1 Department of Geography, Environment and Urban Studies, Temple University, Philadelphia, PA 19122, USA; 2 College of Social Work, University of Tennessee, Knoxville, TN 37996, USA; dcoatswo@utk.edu; 3 Department of Biobehavioral Health, Pennsylvania State University, University Park, PA 16802, USA; mar60@psu.edu; 4 Center for Behavioral Health Research, University of Tennessee, Knoxville, TN 37996, USA; nzaharak@utk.edu (N.Z.); mmason29@utk.edu (M.J.M.); 5 College of Social Work, University of Kentucky, Lexington, KY 40506, USA; a.brown@uky.edu

**Keywords:** depression, mhealth, treatment, intervention, urban, rural, clinical trial, moderation

## Abstract

Depression among young adults represents a growing health problem in the U.S., but access to effective treatment remains a challenge. Mobile health (mHealth) approaches promise to deliver accessible and effective depression treatment; however, questions remain regarding how mHealth depression treatment efficacy may vary geographically based on urban and rural environmental contexts. The present study addresses this knowledge gap by leveraging data from a randomized clinical trial of an mHealth depression treatment called Cognitive Behavioral Therapy-text (CBT-txt) as applied to a sample of 103 U.S. young adults (ages 18–25). Prior research has demonstrated the efficacy of CBT-txt to reduce depressive symptoms. In the present study, we conduct an exploratory, post hoc analysis employing moderated growth curve modeling to investigate whether observed treatment efficacy differed between study participants residing in rural versus urban areas. The findings indicate that CBT-txt treatment effects in terms of reducing depression symptoms were significantly stronger for young adults residing in rural, as compared to urban, regions (β = 13.759, 95% CI = 0.796, 26.723, *p* < 0.038). We speculate that this is because of the lack of mental healthcare resources in rural, as compared to urban areas, as well as the greater level of environmental stressors, such as artificial light and noise, found in cities, which may mitigate treatment effects.

## 1. Introduction

Depression among young adults represents a serious and growing health problem in the U.S., with 17.5% of U.S. young adults (6 million) experiencing a major depressive episode (MDE) in 2023, more than any other age group [1]. The long-term consequences of young adult depression include reduced socioeconomic status, poorer physical health, impaired social functioning, and an increased risk of suicide [2,3]. Unfortunately, access to effective depression treatment remains a challenge, particularly for young adults. Among all age groups, young adults are most likely to recognize the need for mental health treatment but are least likely to receive it [1], due to treatment barriers such as the stigma of acknowledging mental health problems and the high cost of treatment [4,5].

Mobile health (mHealth) approaches, which leverage digital technologies such as mobile phones to deliver behavioral health interventions, show promise in providing accessible and effective depression treatment [6,7]. Such mHealth approaches provide private, accessible, and low-cost treatments, thus reducing common treatment access barriers [8]. Prior studies have shown that cognitive behavioral therapy, an established, evidence-based approach to depression treatment, can be effectively delivered using digital platforms [9,10]. mHealth approaches for depression treatment are particularly appropriate for young adults, who have high usage of mobile phones and text messaging [11] and have shown receptivity to mHealth-delivered behavioral health interventions [12].

An important research gap regarding mHealth depression and other behavioral health treatments concerns the influence of environmental contextual factors on treatment efficacy [13,14,15]. Prior research, for example, has found that mHealth depression treatment effects differed according to neighborhood socioeconomic conditions, where effects were stronger for participants residing in neighborhoods with higher levels of socioeconomic deprivation, even after controlling for individual-level socioeconomic status [16,17]. In the present study, we extend this research to investigate how mHealth depression treatment efficacy may vary according to another key environmental condition of which may impact the etiology and treatment of depression—differences between urban and rural environmental contexts.

A number of prior studies have suggested that excessive sensory stimulation such as the high levels of ambient noise and artificial light found in high-density urban environments may be associated with increased stress and mental disorders such as depression [18,19], while exposure to natural environments may be associated with lower stress and improved mental health [20,21,22]. Despite these potential associations, evidence regarding overall differences in depression prevalence in urban versus rural environments has been inconsistent [23,24,25]. Evidence does suggest, however, that rural residents often have worse mental health outcomes compared to urban residents, as rural residents face increased barriers to mental healthcare, such as high levels of stigma, a lack of transportation options to access treatment, high treatment costs, and a shortage of mental health treatment providers [26].

The objective of this research is to advance understanding of differences between urban and rural areas in terms of mHealth depression treatment efficacy. To this end, we leverage data from a completed pilot study implementing a randomized clinical trial of an mHealth depression treatment called Cognitive Behavioral Therapy-text (CBT-txt) as applied to a sample of 103 young adults (ages 18–25) recruited from across the U.S. Prior research from this trial demonstrated the efficacy of CBT-txt in reducing depressive symptoms across the entire sample [10]. In the present study, we conduct an exploratory, post hoc analysis to investigate whether observed treatment efficacy differed between study participants residing in rural versus urban areas.

## 2. Materials and Methods

### 2.1. The CBT-Txt Depression Treatment

The CBT-txt treatment is an mHealth adaptation of an in-person cognitive behavioral therapy-based treatment [27], for which there is prior evidence of efficacy in reducing depressive symptoms [28]. CBT-txt leverages theoretical mechanisms of behavior change which include increasing behavioral activation (e.g., monitoring daily activities and identifying goals and values), decreasing cognitive distortions (e.g., fixating on the worst possible outcomes), and decreasing perseverative thinking (e.g., excessive rumination) [29]. CBT-txt is delivered to participants via mobile phone SMS text messages. The treatment delivers automated text message ‘conversations’ personalized to the individual based on information about mental health and behavior provided by the participant at enrollment into the study. Messages are tailored based on the participant’s responses regarding their depression symptoms. For example, CBT-txt may ask a participant about their engagement with certain coping skills for which they are provided three choices, where each response choice option activates a different CBT-txt message.

The treatment provides individualized text-based conversations every other day over the course of the intervention. Conversations consist of 474 text messages delivered every other day over an eight-week period. Each week of the intervention focuses on a different topical area: introduction to CBT, automatic thoughts, behavioral activation, automatic thoughts and health, perseverative thinking, cognitive distortions, and social support. For more detailed information on the structure, components, delivery strategy, and theoretical mechanisms of CBT-txt, including sample text messages, the reader is referred to prior publications [10,29].

### 2.2. Study Participant Recruitment and Procedure

Participant criteria for eligibility were (1) age between 18 and 25 years old, (2) moderate or greater depression symptom severity on the Patient Health Questionnaire-9 (PHQ-9) [30], (3) smartphone access, (4) English fluency, (5) no depression treatment over the past three months, and (6) suicidal ideation not endorsed. Advertising on Facebook and Instagram targeting U.S. young adults was conducted from 6 July to 28 August 2022. A website provided more information and screening questions for interested individuals, yielding 103 enrolled study participants who were randomly allocated to the experimental treatment condition (CBT-txt) or a waitlist control condition. Participants who completed the screening and study surveys received $150 in Amazon eGift cards. For further information on participant recruitment, enrollment, data collection, quality control, fidelity, and safety protocol, the reader is referred to prior publications [10,29]. The study was registered at ClinicalTrials.gov (identifier NCT05551702). All procedures were approved by the institutional review board of The University of Tennessee (approval UTK IRB-20-06164-FB).

### 2.3. Measures

The trial lasted three months, with measures collected at four different times: at baseline (enrollment) and at one month, two months, and three months following enrollment. Given that the treatment lasts approximately two months, the one-month data collection represents the halfway point through the treatment, two months represents the end of treatment, and three months represents one month post treatment.

The depression symptoms outcome variable was assessed using the Beck Depression Inventory—II (BDI-II) [31], composed of 21 items, each of which is scored 0–3. Higher scores indicate a higher level of depression severity. The depression symptoms variable was measured for each participant at baseline (α = 0.830) and at one month (α = 0.883), two months (α = 0.916), and three months (α = 0.918) following enrollment.

The time variable indicates when the observation was made: baseline (0), or at one month (1), two months (2), or three months (3) following enrollment.

The condition variable indicates whether the participant was allocated to the treatment group which received CBT-txt (1) or to the waitlist control group (0). The condition variable does not vary over time.

The urban variable indicates whether the participant resided in an urban area (1) or a rural area (0). Urban areas are indicated by U.S. Census Bureau’s urban classification of clusters of census blocks, which reflects areas of high housing unit density, as well as non-residential commercial, industrial, and other developed land uses [32]. Urban areas of greater than 50,000 and 10,000 inhabitants form the core of U.S. federal government-designated metropolitan and micropolitan statistical areas, respectively. Any census blocks which are not classified as within an urban cluster are regarded as rural. We employed the ArcGIS Pro v. 3.2 (ESRI, Redlands, CA, USA) geographic information systems (GIS) software package to geocode each participant’s home address (collected at study enrollment) and encode whether the subject resided in a census-defined urban or rural area. Of the 103 study participants, 86 resided in urban areas and 17 resided in rural areas. The urban variable does not vary over time.

### 2.4. Analytic Plan

We first present descriptive statistics for demographic characteristics at enrollment and for the depression symptoms outcome over time, separately for the treatment and control groups and by participant residence in urban and rural areas. We modeled treatment effects on depression symptom trajectories using growth curve modeling, commonly used to model treatment effects in longitudinal psychological and behavioral assessment data [33]. Growth curve modeling employs random effects, where multiple observations taken over time (level 1) are nested within individuals (level 2) [33,34], as in the present study where surveys are administered at baseline and monthly over a period of three months for each participant. We begin by fitting an unconditional model with time as a linear function and then add time squared to test a quadratic function. Because the model results yield lower Bayesian information criterion (BIC) values for the quadratic function (BIC = 2566.603) as compared to the linear function (BIC = 2595.262), and both the time and time squared variables are significant (*p* < 0.05) in unconditional quadratic function models (β_time_ = −11.040, p_time_ < 0.001; β_time squared_ = 2.287, p_time squared_ < 0.001), both the time and time squared variables are included in further growth curve models.

We first tested for the treatment effect (without yet incorporating urban and rural differences in the treatment effect) by introducing the interaction terms ‘time × condition’ and ‘time squared × condition’ to the growth curve model, which capture the difference between the treatment and control groups in the depression symptoms trajectory over time. We then investigated whether the efficacy of CBT-txt differs between participants residing in urban versus rural areas. We hypothesize that urban versus rural residency will moderate CBT-txt treatment efficacy, such that stronger CBT-txt treatment effects on depression symptoms will be observed among participants residing in rural, as compared to urban, areas. Given that the treatment is intended to reduce depression symptoms over the period of the study, as compared to the control condition, we expect that the difference between the treatment and control groups in the trajectory of depression symptoms over time will be significantly larger for participants residing in rural, as compared to urban, areas.

Following previous research methods [16], we tested for urban versus rural differences in treatment efficacy by introducing the three-way interaction terms ‘time × condition × urban’ and ‘time squared × condition × urban’. A significant (*p* < 0.05) coefficient for either or both of the three-way interaction terms indicates that the hypothesis is supported, i.e., the treatment effect on depression symptoms differs between urban and rural residing participants. As required for the detection of the three-way moderating effect [35], all variables whose products form the three-way interaction (time, time squared, condition, and urban), as well as the derived component two-way interaction terms (time × condition, time squared × condition, time × urban, time squared × urban, and condition × urban), are also included in each model as controls. Graphs of estimated marginal means for treatment and control groups are used to visualize differences in treatment effects for participants residing in urban versus rural areas.

We conducted two sensitivity tests. First, we refitted the models to address the issue of imbalance in the sample, where only 17 out of 103 participants (17% rural) are classified as residing in census-designated rural areas. While this proportion is similar to the United States as a whole (20% rural [36]), we refitted the models to replace the Census Bureau urban/non-urban classification with the National Center for Health Statistics (NCHS) classification [37] of large metropolitan counties (with more than 1 million people) as urban (*n* = 53), and the remaining counties as rural (*n* = 50). Second, we fitted the models to control for individual level confounders, including age (in years), sex (male or female), race (White or non-White), and food assistance (whether the participant had ever received free or reduced lunch or SNAP benefits) as a set of controls for participant demographic characteristics and socioeconomic status. Models were implemented in SPSS v. 28.0.1.0 (IBM Corp., Armonk, NY, USA) using the MIXED command, with a significance threshold of *p* < 0.05.

## 3. Results

Of the 103 participants in the study, the age range was 18–25 (Mean = 22.24, SD = 2.18), 86 (84%) were assigned female at birth, 74 (72%) identified as White race, and 35 (34%) indicated that they had received government food assistance, such as free or reduced lunch or SNAP (Supplemental Nutrition Assistance Program) benefits. Table 1 presents descriptive statistics of the depression symptoms outcome over time, for the treatment and control groups, separately for urban and rural residing participants. Depression symptom values ranged from a high of 34.374 to a low of 13.333. Generally, higher depression symptoms values were observed at baseline and declined over time, except for the rural control group, for which depression symptom values were relatively stable. Depression symptoms declined to a greater extent within the treatment group as compared to the control group, for both urban and rural participants. Among all control/treatment and urban/rural groupings, the greatest decline in depression symptoms occurred in the rural treatment group.

The results from the growth curve models confirm these observed crude trends (Table 2). Consistent with prior research [10], the treatment effects model which does not include moderation by urban and rural participant residency indicates that CBT-txt significantly reduced depression symptoms as compared to the control condition (Model 1; β_time×condition_ = −10.891, 95% CI = −15.748, −6.033, *p* < 0.001). The results from the growth curve models incorporating moderation by urban and rural participant residency indicate our hypothesis is supported; CBT-txt treatment effects on reducing depression symptoms were significantly stronger for young adults residing in rural, as compared to urban, areas (Model 2; β_time×condition×urban_ = 13.759, 95% CI = 0.796, 26.723, *p* = 0.038). The results from the sensitivity analyses where models employed NCHS instead of U.S. Census Bureau urban and rural classification (Model 3; β_time×condition×urban_ = 10.903, 95% CI = 1.030, 20.776, *p* = 0.031) and incorporate participant demographic and socioeconomic controls (Model 4; β_time×condition×urban_ = 13.748, 95% CI = 0.772, 26.724, *p* = 0.038) are consistent with our main results and do not cause us to alter our conclusions.

**Figure 1:**
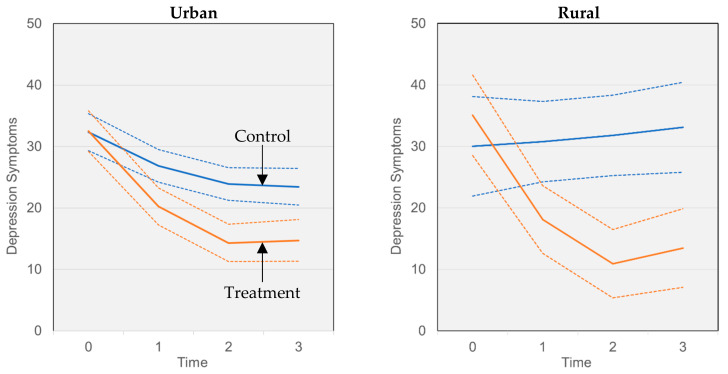
Estimated marginal means (solid lines) from growth models of depression symptoms. Graphs depict trajectories over baseline (0), one-month (1), two-month (2), and three-month (3) timepoints for the treatment (orange) and control (blue) groups, with 95% confidence intervals shown (dashed lines). Separate panels show slopes estimated participants residing in urban (**left**) and rural (**right**) areas.

Figure 1 shows estimated marginal means for depression symptoms over time for the treatment and control groups, separately for urban (left panel) and rural (right panel) residents. Confidence intervals are larger for the rural group, likely due to the smaller sample size. The graphs illustrate that treatment was effective in reducing depression symptoms for residents of both urban and rural areas, as confirmed by pairwise comparisons of estimated depression symptoms between the treatment and control group at each time point, for participants residing in both urban (F_time 0_ = 0.006, *p* = 0.940; F_time 1_ = 10.482, *p* = 0.001; F_time 2_ = 21.941, *p* < 0.001; F_time 3_ = 14.659, *p* < 0.001) and rural (F_time 0_ = 0.908, *p* = 0.341; F_time 1_ = 8.543, *p* = 0.004; F_time 2_ = 23.157, *p* < 0.001; F_time 3_ = 15.905, *p* < 0.001) areas. However, greater differences in the treatment and control groups in terms of the trajectory of depression symptoms over time can be observed for participants residing in rural versus urban areas. For rural areas, treatment resulted in a reduction of approximately 22 points over time on the depression symptoms scale, whereas for urban areas, treatment resulted in a reduction of approximately only 9 points (Table 2, Model 2).

## 4. Discussion

To our knowledge, this is the first study to investigate urban and rural differences in mHealth depression treatment efficacy. Our findings indicate that while CBT-txt reduced depression symptoms for participants residing in both urban and rural areas, among this sample of 103 young adults, the difference in the trajectory of depression symptoms between those receiving CBT-txt versus the control condition was greater among rural participants. We speculate that greater efficacy in CBT-txt treatment observed in rural areas may be associated with the lack of mental healthcare resources [26,38], as compared to more readily available resources in urban areas. Rural residents who select into depression treatment may be more receptive to treatment messaging and motivated to adhere to treatment given otherwise limited treatment options, thus enhancing the observed efficacy of mHealth treatment approaches in rural regions. While speculative, this interpretation is consistent with previous findings which found stronger treatment efficacy in higher deprivation neighborhoods (independent of whether those neighborhoods were in urban or rural areas) [16,17], whose residents often have poorer access to mental healthcare and mental health-promoting amenities relative to those residing in more socioeconomically advantaged neighborhoods [39,40,41].

As additional post hoc sensitivity analyses to investigate the effects of access to mental healthcare and area-level socioeconomic status on depression symptoms, we refitted the models to control not only for individual-level age, sex, race, and socioeconomic status via receiving food assistance but also the number of mental health providers within the residential county, as derived from the U.S. Centers for Medicare and Medicaid Services’ National Provider Identifier [42] as well as the Area Deprivation Index (ADI), a well-established measure of neighborhood (census tract)-level socioeconomic status composed of multiple census variables [43,44]. We did not find that either the number of mental health providers or the ADI had a significant effect on depression symptoms (β_mental health providers_ = −0.0001, 95% CI = −0.001, 0.0004, *p* = 0.585; β_ADI_ = −0.026, 95% CI = −0.090, 0.038, *p* = 0.425). In addition, inclusion of these variables as covariates yielded results consistent with our main findings regarding observed significant differences in CBT-txt treatment efficacy between participants residing in urban versus rural areas (β_time×condition×urban_ = 16.116, 95% CI = 3.567, 28.664, *p* = 0.012). We acknowledge these results do not preclude the additional moderating effects of either mental health provider access or area-level socioeconomic status on treatment efficacy and recommend this as an important topic of future research with larger sample sizes, which may better support multiple simultaneous tests of moderation.

The observed difference in CBT-txt treatment efficacy between participants residing in urban versus rural areas may also be due to the greater level of exposure to environmental stressors in cities. Prior research has found that several physical environmental characteristics more common in urban regions may negatively influence mental health, such as exposure to artificial light [45], air pollution [46], ambient noise [47], and crowding [48], characteristics which are theorized to negatively influence mental health outcomes via environmental impacts on neurobiological function [19]. Conversely, environmental characteristics more commonly found in rural areas, such as exposure to natural landscapes with greater vegetation or tree cover, are associated with positive affect, improved memory, and elevated mood [20,22,49]. These positive mental health effects of exposure to natural environments are theorized to operate via the restoration of attention through relaxation of neurocognitive function [50] as well as psychological stress reduction [51]. Urban environmental stressors may mitigate receptivity to CBT-txt treatment messaging, thus resulting in weaker treatment efficacy in urban, as compared to rural, areas. Although neighborhood deprivation can also be considered an environmental stressor, there may be something unique about such environmental stressors in urban areas that weakens the effect of treatment. While the present research does not provide evidence for whether these environmental characteristics do, in fact, explain the observed differences in treatment efficacy between residents of urban and rural regions, it does highlight the disentangling moderating, mediating, and interacting effects of urban versus rural setting with neighborhood deprivation and other environmental characteristics as an important topic of future research.

We acknowledge that the study sample size is relatively small (*N* = 103), with the sample of rural residents in our main analysis numbering only 17 participants, and we therefore consider our findings exploratory, with unknown generalizability to other populations outside the sample. In addition, while the BDI-II is a well-established instrument for measuring depression symptoms, reliability could be enhanced by the use of other depression measures. Nonetheless, our analysis benefits from several advantages which lend credence to the results: (1) a sample that is, while not nationally representative, national in scope, with participants residing in 34 separate states; (2) an experimental, longitudinal research design that leverages data from a randomized clinical trial, supporting causal inference; (3) sensitivity analysis results that are highly consistent with the main findings and that employ a different measure of urban and rural classification with a more balanced sample and a larger number of rural participants, as well as demographic and socioeconomic status controls; and (4) the consistency of the results with established theoretical mechanisms of depression and depression treatment associated with urban and rural environments. In addition, while it is well-known that confidence intervals provide evidence of post hoc statistical power [52], as provided in the present manuscript, we note that our study also can provide guidance for sample size in future prospective studies of urban–rural differences in mHealth depression treatment. A post hoc multilevel modeling power analysis [53] of the observed urban–rural moderating effect size yielded a sample size of 188 (level 2) participants to achieve 0.80 power at *p* < 0.05, an estimate which may guide future prospective research designs for randomized controlled trials investigating urban and rural differences in depression treatment efficacy.

## 5. Conclusions

This research reinforces the potential of mHealth treatments to address the rising prevalence of depression among young adults, while highlighting urban and rural differences in mHealth treatment efficacy. Our finding that mHealth depression treatment efficacy may be stronger for participants residing in rural, as compared to urban, areas suggests that certain characteristics of urban and rural environments can either suppress or reinforce mHealth depression treatment mechanisms and outcomes. Future research employing larger sample sizes, with a priori study designs explicitly addressing urban and rural participant residency, are needed to better understand which specific urban and rural environmental characteristics may influence treatment efficacy, and how urban and rural environmental conditions may impact treatment outcomes via targeted treatment mechanisms. The overall effectiveness of a treatment in a public health setting also depends on treatment adoption by those in need, including the use of direct-to-consumer marketing to target patients and their caregivers [54].

## Figures and Tables

**Table 1:** Descriptive statistics for depression symptoms at baseline and one month, two months, and three months after enrollment, for urban and rural residing participants. Means are reported with standard deviations in parentheses.

	Urban		Rural	
Time	Control Group	Treatment Group	Control Group	Treatment Group
Baseline	32.744 (8.876)	32.500 (8.645)	30.000 (11.726)	34.375 (10.295)
1 month	26.561 (8.826)	18.414 (9.171)	30.000 (9.592)	16.400 (11.404)
2 months	23.700 (11.660)	14.807 (8.708)	32.571 (12.000)	13.333 (11.576)
3 months	23.262 (9.976)	14.065 (11.087)	32.857 (6.670)	13.333 (12.124)

**Table 2:** Results of growth curve models of the CBT-txt treatment effect and the moderating effect of urban versus rural on the treatment effect. Unstandardized coefficients and significance are reported, with 95% confidence intervals in parentheses. Significant coefficients (*p* < 0.05) are in bold.

Independent Variables	Model 1 Treatment Effect	Model 2 Moderation by Urban (Census Classification)	Model 3 Moderation by Urban (NCHS Classification)	Model 4 Moderation by Urban (Census) w/Controls
Time	**−5.191, *p* < 0.001** **(09.248, 2.590)**	0.612, *p* = 0.895 (−8.526, 9.750)	−2944, *p* = 0.270 (−8.186, 2.297)	0.644, *p* = 0.890 (−8.502, 9.791)
Time Squared	**1.155, *p* = 0.031** **(0.106, 2.205)**	0.140, *p* = 0.922 (−2.660, 2.940)	0.325, *p* = 0.697 (−1.317, 1.968)	0.132, *p* = 0.926 (−2.670, 2.935)
Condition (Ref = Control)	0.774, *p* = 0.711 (−3.337, 4.885)	−0.607, *p* = 0.877 (−8.333, 7.119)	−0.305, *p* = 0.919 (−6.177, 5.567)	−4.643, *p* = 0.387 (−5.905, 15.192)
Urban		2.332, *p* = 0.596 (−6.312, 10.976)	−1.317, *p* = 0.653 (−7.085, 4.452)	2.481, *p* = 0.581 (−6.353, 11.314)
Time × Condition	**−10.891, *p* < 0.001** **(−15.748, −6.033)**	**−22.425, *p* < 0.001** **(−34.259, −10.590)**	**−16.154, *p* < 0.001** **(−23.111, −9.197)**	**−22.400, *p* < 0.001** **(−34.245, −10.556)**
Time Squared × Condition	**2.387, *p* = 0.003** **(0.842, 3.931)**	**4.733, *p* = 0.012** **(1.069, 8.396)**	**3.803, *p* < 0.001** **(1.594, 6.011)**	**4.718, *p* = 0.012** **(1.052, 8.384)**
Time × Urban		−7.383, *p* = 0.139 (−17.177, 2.411)	−5.056, *p* = 0.143 (−11.829, 1.717)	−7.406, *p* = 0.138 (−17.208, 2.395)
Time Squared × Urban		1.129, *p* = 0.461 (−1.885, 0.006)	1.417, *p* = 0.192 (−0.714, 3.548)	1.139, *p* = 0.458 (−1.877, 4.155)
Condition × Urban		−0.002, *p* = 0.974 (−0.146, 4.144)	2.013, *p* = 0.632 (−6.257, 10.283)	−4.448, *p* = 0.448 (−15.986, 7.091)
Time × Condition × Urban		**13.759, *p* = 0.038** **(0.796, 26.723)**	**10.903, *p* = 0.031** **(1.030, 20.776)**	**13.748, *p* = 0.038** **(0.772, 26.724)**
Time Sq. × Condition × Urban		−2.833, *p* = 0.168 (−6.869, 1.202)	−2.942, *p* = 0.066 (−6.075, 0.190)	−2.827, *p* = 0.169 (−6.866, 1.211)
Age (years)				0.307, *p* = 0.430 (−0.461, 1.075)
Sex (Ref = Male)				0.785, *p* = 722 (−3.572, 5.143)
Race (Ref = White)				−1.317, *p* = 0.496 (−5.141, 2.507)
Food Assistance (Ref = Received)				0.701, *p* = 691 (−2.790, 4.191)

## Data Availability

The data presented in this study are not publicly available due to privacy restrictions.
